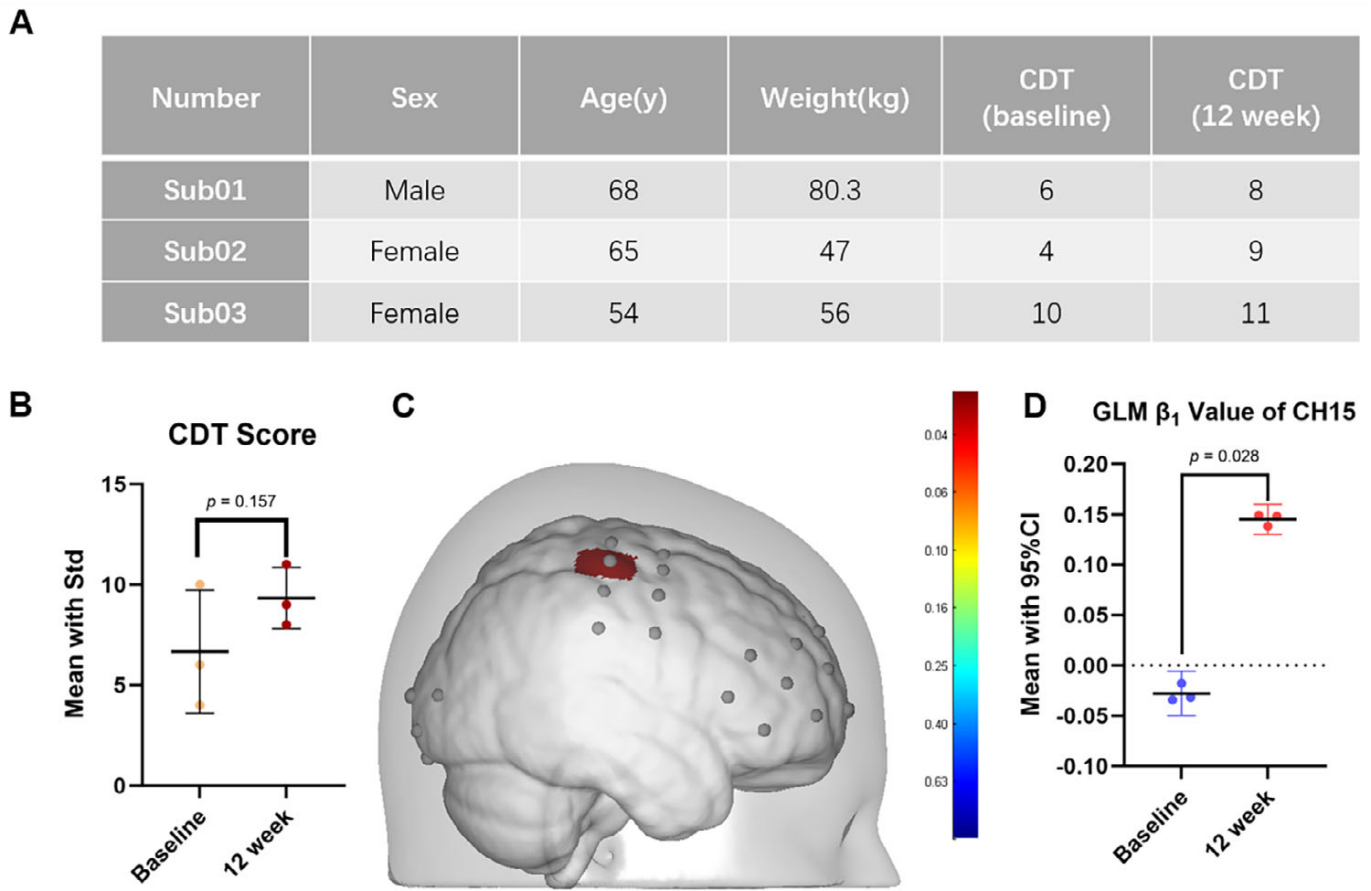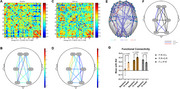# Functional Near‐Infrared Spectroscopy Reveals Lecanemab‐Induced Neural Changes During the Clock Drawing Test in Alzheimer's Disease

**DOI:** 10.1002/alz70859_102585

**Published:** 2025-12-25

**Authors:** Wenbo Zhang, Ming Chen, Qi Tian, Weihua Yu, Yang Lü

**Affiliations:** ^1^ The First Affiliated Hospital of Chongqing Medical University, Chongqing, Chongqing China; ^2^ Chongqing Medical University, Chongqing, Chongqing China

## Abstract

**Background:**

Lecanemab, a monoclonal antibody targeting amyloid‐beta (Aβ) aggregates, has shown efficacy in reducing Aβ burden in Alzheimer’s disease (AD). However, its impact on brain function remains understudied. We employed functional near‐infrared spectroscopy (fNIRS) during the Clock Drawing Test (CDT) to examine potential changes in brain function following lecanemab treatment.

**Method:**

Three patients with clinically diagnosed AD were recruited from the Memory Clinic of The First Affiliated Hospital of Chongqing Medical University. Each patient underwent fNIRS assessments during a CDT paradigm (task + 20 s rest blocks) at baseline and after 12 weeks of lecanemab therapy. Hemodynamic responses were analyzed using a generalized linear model (GLM) to extract β1 values. We further assessed functional connectivity (FC) among predefined regions of interest (ROIs). A paired t‐test was conducted to compare CDT performance (15‐point CLOX system) and fNIRS‐derived metrics before and after treatment.

**Result:**

Two patients reported no adverse events; one experienced a mild headache following the first infusion. CDT scores did not show a statistically significant improvement after 12 weeks (*p* = 0.157). In contrast, fNIRS data revealed a significant increase in β1 values at channel 15 (right primary somatosensory cortex) (*p* < 0.05, FDR corrected). ROI‐based FC analysis demonstrated enhanced connectivity between the right frontal (F.R) and left occipital (O.L) regions, as well as between the right parietal (P.R) and right occipital (O.R) regions (both *p* < 0.05, FDR corrected). Conversely, FC between the left frontal (F.L) and right parietal (P.R) regions decreased (*p* < 0.05, FDR corrected).

**Conclusion:**

Lecanemab therapy may induce notable changes in brain functional connectivity and neural activation patterns in AD, as evidenced by fNIRS during the CDT. The observed increase in frontal–occipital FC may indicate improved network coordination, whereas the decreased parietal–frontal connectivity could reflect compensatory reorganization. These findings underscore the potential of fNIRS for monitoring treatment‐related neuronal changes in AD and warrant validation in larger cohorts.